# Chaetotaxy of the fourth larval stage of *Pintomyia longiflocosa,* a primary vector of cutaneous leishmaniasis in Colombia

**DOI:** 10.7705/biomedica.7124

**Published:** 2024-11-06

**Authors:** Sergio Méndez-Cardona, María Cristina Carrasquilla, Camila González, Erika Santamaría

**Affiliations:** 1 Grupo de Entomología, Instituto Nacional de Salud, Bogotá, D. C., Colombia Instituto Nacional de Salud Bogotá D. C. Colombia; 2 Centro de Investigaciones en Microbiología y Parasitología Tropical, Universidad de los Andes, Bogotá, D. C., Colombia Universidad de los Andes Universidad de los Andes Bogotá D. C Colombia

**Keywords:** Psychodidae, insect vectors, leishmaniasis, cutaneous, larva, microscopy, electron, classification, Psychodidae, insectos vectores, leishmaniasis cutánea, larva, microscopía electrónica, clasificación

## Abstract

**Introduction.:**

*Pintomyia (Pifanomyia) longiflocosa* is an endemic species from Colombia, found between the central and eastern Andes, and reported as one of the primary vectors of cutaneous leishmaniasis in coffee-growing zones of the country. This species is classified in the Townsendi series and can only be identified by the morphology of the male adults.

**Objective.:**

To determine the potential use of the fourth larval stage of the vector *Pi. longiflocosa* in morphological taxonomy based on the description of its chaetotaxy.

**Materials and methods.:**

*Pintomyia longiflocosa* adults were captured in Campoalegre, Huila, and reared in the Entomology Laboratory at the Colombian *Instituto Nacional de Salud.* To identify the setae found in each corporal segment, 15 fourth-instar larvae were mounted on microscope slides using Canadian balm after being cleared with 10 % potassium hydroxide and saturated phenol. Additionally, five specimens were prepared for their observation by scanning electron microscopy.

**Results.:**

Based on the description of *Pi. longiflocosa,* we established that all species of the subgenus *Pifanomyia* so far described have the same antennal morphology and clavate setae along their body. However, various setae present in *Pi. longiflocosa* are absent in *Pi. youngi,* suggesting differences among the larvae of the Townsendi series.

**Conclusions.:**

These results support the potential importance of morphological characters from the fourth larval instar, such as antennal morphology and chaetotaxy, specifically in closely related species that are cryptic in their adult stages.

## Resumen

**Introducción.:**

*Pintomyia (Pifanomyia) longiflocosa* es una especie endémica de Colombia, encontrada entre la cordillera central y oriental, y reportada como uno de los principales vectores de leishmaniasis cutánea en las zonas cafeteras del país. Esta especie se clasifica en la serie Townsendi y solo se puede identificar por la morfología de los machos adultos.

**Objetivo.:**

Determinar el uso potencial del cuarto estadio larvario del vector *Pi. longiflocosa* en la taxonomía morfológica a partir de la descripción de su quetotaxia.

**Materiales y métodos.:**

Se capturaron adultos de *Pintomyia longiflocosa* en Campoalegre, Huila y se mantuvieron en el Laboratorio de Entomología del Instituto Nacional de Salud de Colombia. Para identificar las setas encontradas en cada segmento corporal, se montaron 15 larvas de cuarto estadio en portaobjetos de microscopio utilizando bálsamo de Canadá después de ser aclaradas con hidróxido de potasio al 10 % y fenol saturado. Además, se prepararon cinco especímenes para observarlos por microscopía electrónica de barrido.

**Resultados.:**

Basados en la descripción de *Pi. longiflocosa,* se estableció que todas las especies del subgénero *Pifanomyia* descritas hasta ahora tienen la misma morfología de las antenas y presentan setas clavadas a lo largo del cuerpo. Sin embargo, varias setas presentes en *Pi. longiflocosa* están ausentes en *Pi. youngi,* lo que sugiere diferencias entre las larvas de la serie Townsendi.

**Conclusiones.:**

Estos resultados respaldan la importancia potencial de los caracteres morfológicos del cuarto estadio larval, como la morfología de las antenas y la quetotaxia, específicamente en especies estrechamente relacionadas, crípticas en sus etapas adultas.

The leishmaniases are a group of neglected tropical diseases caused by *Leishmania* parasites, transmitted by the bite of females of the subfamily Phlebotominae (Diptera: Psychodidae). In Colombia, this disease is endemic in almost the entire territory located below an elevation of 1,750 MASL, presenting the three main clinical forms of the disease, with cutaneous leishmaniasis having the highest incidence ^1^. According to the Colombian epidemiological surveillance system (SIVIGILA), between 2008 and 2018, 108,718 cases of cutaneous leishmaniasis were reported, corresponding to 98.5% of the total.

Regarding the vectors, only nine of the 163 sand fly species recorded in Colombia are proven vectors of *Leishmania* parasites causing cutaneous leishmaniasis ^2^. One of the most important species is *Pintomyia longiflocosa* (Osorno-Mesa, Morales, Osorno & Hoyos; 1970), which was involved as vector in two epidemic outbreaks of cutaneous leishmaniasis in Colombia: one in the municipalities of Neiva, Baraya, and Tello in the department of Huila, between 1993 and 1996 ^3^, and the most recent in Chaparral (Tolima), between 2004 and 2005 ^4^^-^^7^. This sand fly species has also been implicated as the most probable vector in leishmaniasis epidemic outbreaks in Planadas (Tolima) ^8^, and Ábrego (Norte de Santander) ^9^.

*Pintomyia longiflocosa* is endemic to Colombia and is distributed between the Central and Eastern Cordilleras from 900 to 2,100 MASL. It is particularly abundant between 1,500 and 1,700 MASL in coffee-growing areas ^3^^,^^10^. This species belongs to the subgenus *Pifanomyia,* Townsendi series ^11^, of which at least four other species have been implicated in *Leishmania* transmission, causing the cutaneous clinical form ^12^. In this series, adult females are isomorphic, and their morphological identification is based exclusively on adult male characters, so species delimitation has been resolved mainly through morphometric and molecular methods ^11^^,^^12^.

As an alternative to identification in cryptic species, the study of the morphology of immature stages has shown potential in the discovery of new useful taxonomic characters, like in the case of phlebotomine sand flies, where chaetotaxy of the fourth larval stage has been explored with a greater interest ^13^. Nonetheless, in the *Pifanomyia* subgenus, the few descriptions available have limited understanding of the taxonomical value of larval characters ^12^. The fourth larval stage of only five species from this subgenus: *Pi. ovallesi, Pi. verrucatum, Pi. evansi, Pi. serrana,* and *Pi. youngi*^14^^-^^17^ have been partially or fully described, with the latter being the only described species in the Townsendi series.

Considering the importance of vector identification in the study of leishmaniasis transmission foci ^18^ and that larvae morphological characters could help in the differentiation of species in the *Pifanomyia* subgenus, the objective of this work was to describe the chaetotaxy of the fourth larval stage of the vector *Pi. longiflocosa,* to compare it to previous descriptions of closely related species and to determine the potential use of this stage in morphological taxonomy.

## Materials and methods

The sampling of *Pi. longiflocosa* adults was carried out using a Shannon light trap in a forest located in the dispersed rural settlement of Venecia (2° 39' 47" N; 75° 14' 31" W), in Campoalegre, department of Huila, at an elevation range between 1,400 and 1,700 MASL. The annual precipitation in this area is approximately 1,000 mm, which occurs bimodally with low rainfall periods in the first two months of the year and mid-year. The land in this area is used mainly for the cultivation of unshaded coffee ^19^. A previous study in this locality revealed that approximately 90% of the sand flies were *Pi. longiflocosa*^20^.

All collected phlebotomine sand flies were placed in a muslin cage (15 x 15 x 15 cm) for mating and blood feeding of the females. Afterward, 15 females and 15 males were transferred to rearing containers (150 cm^3^ transparent polystyrene cups previously prepared with plaster of Paris) ^21^ with *ad libitum* water and sucrose solution supply. Specimens were transported in a styrofoam box to the Entomology Laboratory of the *Instituto Nacional de Salud* in Bogotá.

After oviposition, the adults were removed and stored in 70% ethanol while the immature stages were raised according to previously established methodologies proposed by Ferro *et al.*^22^^)^ and Modi and Tesh ^21^. The adults were processed following the clearing procedure described by Young and Duncan ^23^, and all were identified as *Pi. longiflocosa* according to the keys by Galati ^11^.

Between 45 and 60 days after oviposition, when all larvae had reached their fourth stage, larvae were stored in 70% ethanol. Twenty of these larvae were randomly selected for making the morphological description (15 for observation using an optical microscope and five for scanning electron microscopy).

For optical microscopy observation, larvae were mounted following the methodology described by Ferro *et al.*^24^: Specimen clearing was performed by placing the larvae in 10% potassium hydroxide (KOH) overnight and then in saturated phenol for 30 minutes. Following this, the larvae were placed dorsally or laterally on a microscope slide with a drop of a mixture of Canadian balm (one part) and saturated phenol (three parts), allowing the mixture to dry overnight before covering the specimens with a cover slip and an additional drop. The description of setae position and size on the larvae was illustrated and labeled based on the nomenclature proposed by Forattini ^25^. For setae not named in the Forattini nomenclature, we used the system of Arrivillaga *et al.*^26^.

The larvae examined by scanning electron microscopy at the Microscopy Center - Microcore (https://microcore.uniandes.edu.co/en/) of the *Universidad de los Andes* were mounted on a copper wire and covered with colloidal gold twice in such a way to observe different body axes of the same individual.

## Results

### Head

**Figure 1:**
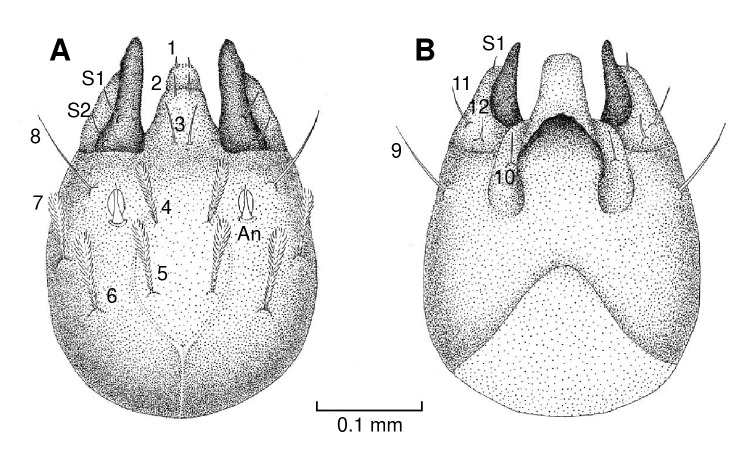
Diagram of the chaetotaxy of the head of *Pintomyia longiflocosa.* **A)** Dorsal view. **B)** Ventral view.

**Figure 2:**
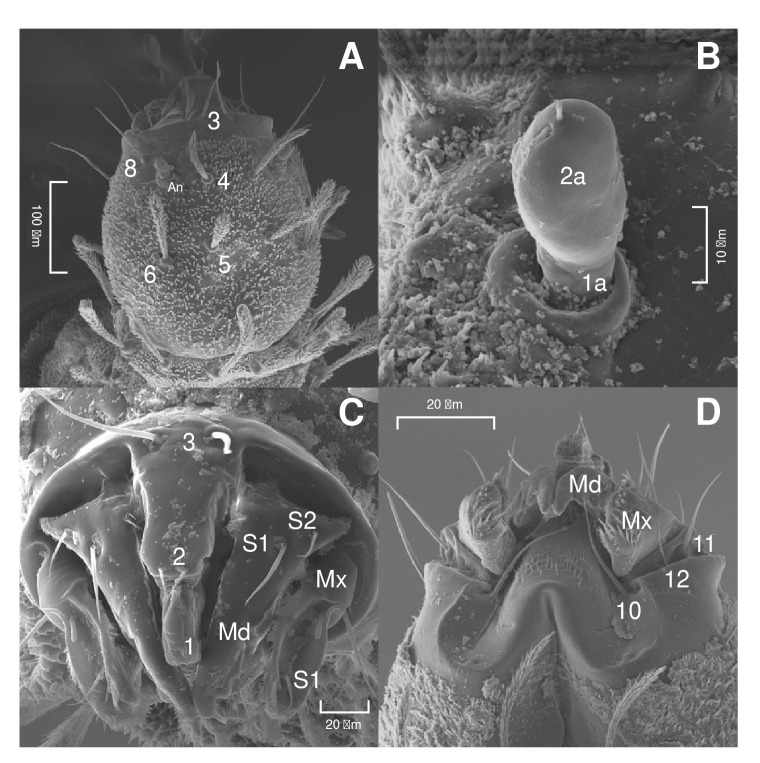
Scanning electron microscopy of the head of *Pintomyia longiflocosa.* **A)** Dorsal view of the head showing the setae positions 5 and 6 on the same horizontal plane. **B)** Antennal segments. **C)** Front view of the oral apparatus. **D)** Ventral view of mouthparts showing setae 10, 11 and 12.

The head is ovoid-shaped (figure 1 and 2A) with short antennae inserted on tubercles. The first antennal segment is shorter, and the second is elliptical with a groove along the midline. The antenna has a terminal spiniform appendage (figure 2B).

On the dorsal axis (figure 1A), setae 1 and 2 are spiniform, while setae 3 and 8 are filiform with a broad base. Setae 4, 5, 6, and 7 are clavate and feathery, inserted on tubercles. Setae 5 and 6 are positioned along the same horizontal plane on the head (figure 2A). The mandibular setae, S1 and S2, are spiniform (figure 2C). On the ventral axis (figure 1B), setae 9 and 10 are filiform. Maxillary setae 11, 12, and S1 are spiniform (figure 2D).

**Figure 3:**
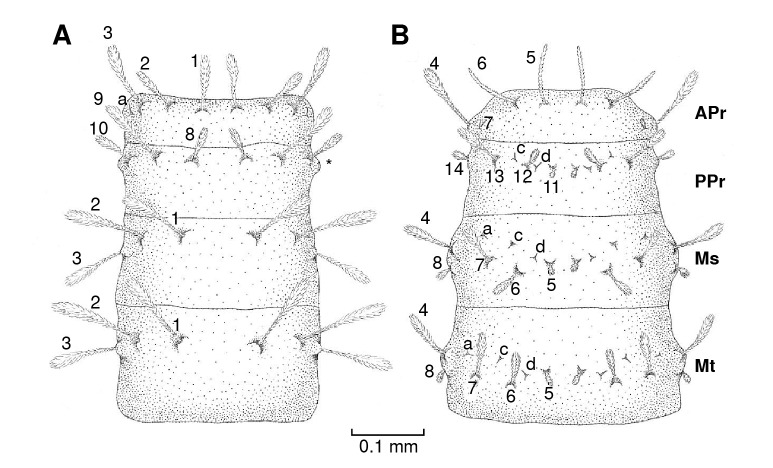
Diagram of the thorax chaetotaxy of *Pintomyia longiflocosa.* **A)** Dorsal view. **B)** Ventral view. The asterisk (*) indicates the position of the anterior spiracle.

### Anterior prothorax

**Figure 4:**
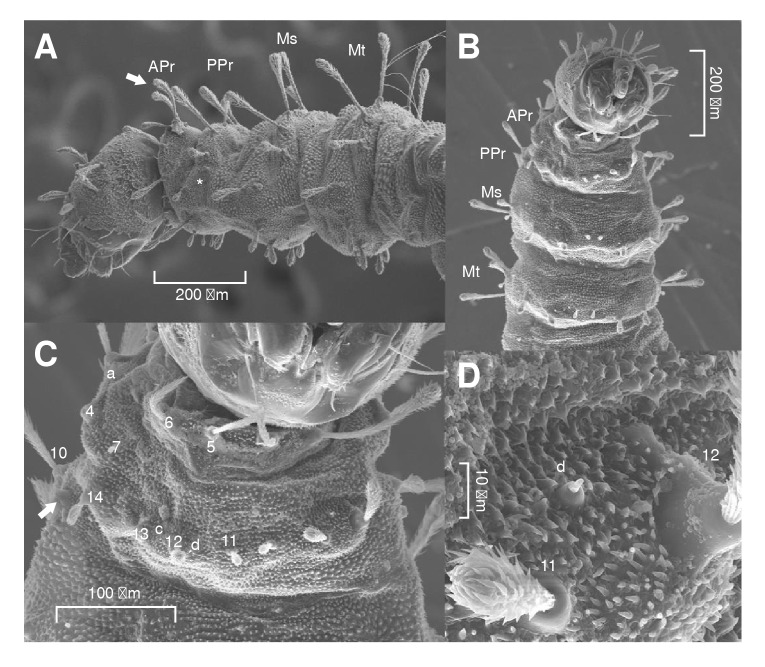
Scanning electron microscopy of the thorax of ***Pintomyia longiflocosa. ***A)****** Lateral view of the head and thoracic segments. The arrow highlights the clavate morphology of the setae. The asterisk (*) indicates the position of the anterior spiracle. **B)** Ventral view of the thorax. **C)** Ventral view of the prothorax. The arrow points to the anterior spiracle. **D)** Detail of the prothorax showing setae d between 11 and 12.

On the dorsal axis (figure 3A), setae 1, 2, and 3 are long, clavate, feathery, and inserted on tubercles (figure 4A). Seta a is spiniform. On the ventral axis (figure 3B), the setae are similar in size. Setae 4, 5, and 6 are feathery, with setae 4 being clavate while 5 and 6 are thin and of uniform thickness. Seta 7 is also feathery, but of a reduced size, giving it a globular appearance.

### Posterior prothorax

On the dorsal axis (figure 3A), setae 8, 9, and 10 are long, clavate, feathery, and inserted on tubercles. The pair of anterior spiracles are small and are located on the lateral margin (figure 4A). On the ventral axis (figure 3B), setae 11, 12, 13, and 14 are feathery and variable in size but noticeably shorter than setae on the dorsal axis (figure 4B). Setae c and d are spiniform (figure 4C and figure 4D).

### Mesothorax and metathorax

On the dorsal axis (figure 3A), setae 1, 2, and 3 are feathery. On the ventral part (figure 3B), setae 4, 5, 6, 7, and 8 are small and feathery (figure 4B). Setae a, c and d are spiniform.

### Abdominal segments I-VII

**Figure 5:**
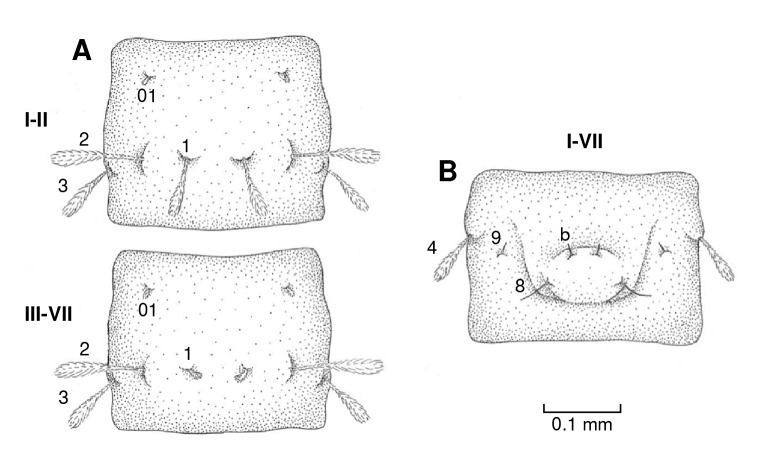
Diagram of the abdomen chaetotaxy of *Pintomyia longiflocosa,* segments l-VII. **A)** Dorsal view. **B)** Ventral view.

**Figure 6:**
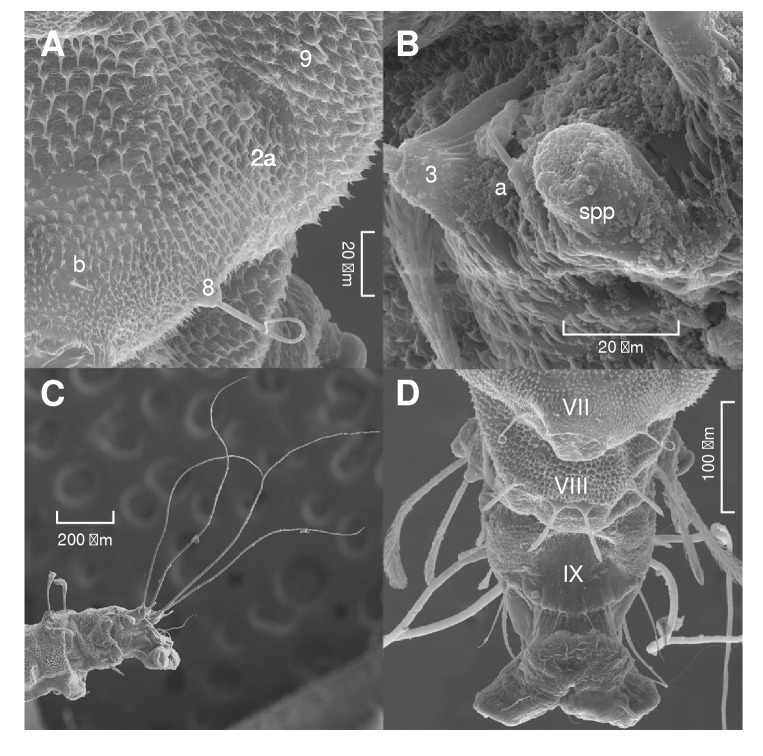
Scanning electron microscopy of the abdomen of *Pintomyia longiflocosa.* **A)** Pseudopodium detail showing setae 8, 9, and b. **B)** Posterior spiracle (spp) and seta a in the eighth abdominal segment. **C)** Lateral view of segments VII to IX showing the total length of setae 2a and 2b. **D)** Ventral view of segments VII-IX.

On the dorsal axis (figure 5A), setae 01, 1, 2, and 3 are clavate and feathery. Seta 01 is small and found close to the base of the segment between setae 1 and 2. Starting on the third abdominal segment, seta 1 is smaller. On the ventral axis, segments I-VII all have a pseudopodium covering most of the area (figure 5B). Seta 4 is feathery, and setae 8, 9, and "b" are spiniform (Figure 6A).

### Abdominal segment VIII

**Figure 7:**
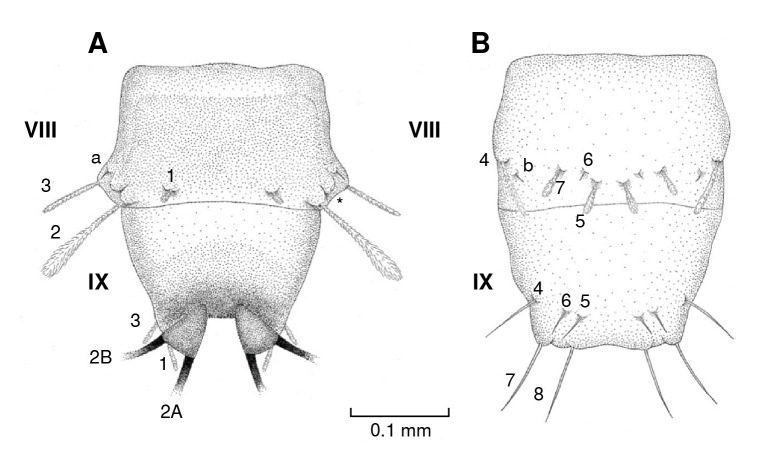
Diagram of the abdomen chaetotaxy of *Pi. Longiflocosa,* segments VIII and IX. **A)** Dorsal view. **B)** Ventral view. The asterisk (*) indicates the position of the posterior spiracle.

On the dorsal axis (figure 7A), setae 1, 2, and 3 are feathery and attached to tubercles, but seta 3 has a constant width, unlike setae 1 and 2. Seta a is adjacent to the posterior spiracle and is small and spiniform (figure 6B). On the ventral axis (figure 7B), setae 4, 5, and 7 are feathery, while 6 and b are spiniform.

### Abdominal segment IX

This segment has an intensely pigmented dorsal tergal plate (figure 7A). Setae 1 and 3 are feathery with uniform thickness. Setae 2A and 2B are pigmented and thread-like (figure 6C). On the ventral axis, abdominal segment IX ends in two anal tubercles (figure 6D), setae 4, 5, 6, 7, and 8 are spiniform (figure 7B).

## Discussion

The identification of sand fly species has primarily focused on the adult stage. Accordingly, the morphology of the larvae has not been widely studied, with only 92 of more than 500 neotropical sand fly species fully or partially described ^11^^,^^13^. The difficulty in finding sand fly larvae in their natural habitat and the lack of success in breeding sand flies explains the dearth of studies on this developmental stage. As a contribution to this field, through observations on optical and scanning electron microscopy, the chaetotaxy of the fourth larval stage of the vector *Pi. longiflocosa* is described for the first time.

### Comparison of the description of Pifanomyia longiflocosa description with closely related species of the subgenus Pifanomyia

When comparing our observations with previous descriptions of species in the subgenus *Pifanomyia,* it was apparent that they all share the same antennal morphology and have clavate setae along their body. According to the grouping by Leite and Williams ^27^, *Pi. longiflocosa* and the other species of the subgenus *Pifanomyia* described so far are in group 4 of the neotropical sand fly larvae classification as they present an annular antennal tubercle, a short basal segment, and an ovoid distal segment (figure 2B). This character makes it possible to differentiate the larvae of the subgenus *Pifanomyia* from those of the subgenus *Pintomyia.* Descriptions of the head of *Pi. fischeri* and *Pi. damascenoi*suggest that the antennal morphology of those species corresponds to group 2, characterized by an antenna tubercle in the shape of a truncated cone, a short basal segment, and a banana-shaped distal segment ^25^^,^^27^.

In the subgenus *Pifanomyia,* the chaetotaxy of the fourth larval stage had been described for a small number of species (five of 74 species), and the absence of commonalities amongst those descriptions indicated no known useful characters to differentiate species. Through comparison with those previous descriptions, it was possible to establish morphological characters supporting series differentiation and species identification within the series. The Serrana series, represented by *Pi. serrana,* has the greatest differences from the other species. On abdominal segment III, the dorsal seta 1 is subequal to seta 2 and, therefore, does not present the same size reduction seen on *Pi. longiflocosa* and the other *Pifanomyia* species. However, the incomplete description of this species and the lateral view of some illustrations hinder the establishment of additional characters because so far chaetotaxy is based on a common nomenclature ^14^.

As mentioned above, the species described of the series Townsendi and Evansi share a common trait of size reduction of dorsal seta 1, starting on abdominal segment III. Nevertheless, the series can be differentiated by the relative size of ventral seta 4 on abdominal segment VII. For species of the Evansi series (Pi. *evansi* and *Pi. ovallesi),* seta 4 is distinctly shorter than seta 5, but nearly the same size as seta b ^14^^,^^16^. On the other hand, seta 4 of the species in the Townsendi series *(Pi. longiflocosa* and *Pi. youngi)* is longer than seta 5. Based on the species descriptions of the Evansi series, it could be proposed that in the absence of abdominal segment VII, seta a in *Pi. ovallesi* can be used to differentiate from *Pi. evansi.* It is also worth mentioning that for *Pi. evansi,* seta 8 of the head capsule is closer to the middle of the dorsum than any other *Pifanomyia* species. However, as is the case with *Pi. serrana,* the incomplete description of *Pi. ovallesi* limits the possibility of further comparison ^14^.

### Comparison of Pifanomyia longiflocosa description with closely related species of the Townsendi series

*Pifanomyia youngi* is the only species of the Townsendi series with the chaetotaxy described for the fourth larval stage ^16^. The setae arrangement of the fourth larval stage of *Pi. longiflocosa* is very similar to *Pi. youngi.* They have the same position and size patterns along the body but with some specific differences. In the head of *Pi. youngi,* seta 2 is absent as are setae a, c, and d in the segments of the thorax. Additionally, in *Pi. youngi,* seta 6 is observed on the metathorax, but not on the mesothorax. On the abdomen of *Pi. youngi,* seta 01 is absent, as is seta a, usually accompanying the posterior spiracle on the eighth segment.

In the description of *Pi. youngi* and *Pi. evansi*^16^^,^^17^, the setae described as a and b are found on the maxillae and not on the mandibles, as seen in *Pi. longiflocosa.* It is suggested that the position of these setae was an error when illustrating chaetotaxy because an equivalent pair of setae appears on the dorsal part of the mandibles of *Pi. longiflocosa, Lutzomyia longipalpis* and *Migonemyia migonei*^13^^,^^27^^,^^28^. Additionally, it is unlikely that a pair of dorsal setae is present in the maxillae, given that the largest surface area of this part of the oral apparatus is oriented laterally. Furthermore, setae 11 and 12 are in this lateral position, reinforcing this observation.

The above clarification is relevant when considering that, at the time chaetotaxy of *Pi. youngi* was described, setae a and b were highlighted as novel traits regarding the Forattini ^25^ classification system ^16^. However, the illustrations accompanying Forattini's nomenclature show the mandibular setae but are unnumbered. In 1999, Arrivillaga *et al.*^26^ named the equivalent of setae a and b as mandibular S1 and S2 and established the presence of three setae in the maxillae, corresponding to setae S1, S2, and S3. The maxillary S1 seta was also highlighted in *Pi. youngi* as an unmarked seta in the system of Forattini ^25^, which again had been illustrated but not numbered.

The shared patterns found amongst the species of subgenus *Pifanomyia* not only bring additional support for their taxonomy but may also be indicative of similar conditions in their breeding sites as the relative size of larval structures has been previously associated with specific characteristics, such as breeding site depth ^29^^,^^30^. The breeding sites of *Pi. longiflocosa* have not been explored, but the similarities found in the chaetotaxy across the subgenus could optimize future searches based on the previous characterization of the microhabitat where the immature stages of *Pi. evansi, Pi. ovallesi* and *Pi. serrana* are found in Colombia ^31^.

In conclusion, chaetotaxy description of the fourth larval stage of *Pi. longiflocosa* and the differences identified with other species support the notion that this species' larvae do possess diagnostic characters useful for species differentiation within the *Pifanomyia* subgenus. This fact is particularly important for species identification of the Townsendi series, considering that the adult females are isomorphic, but the larvae of *Pi. youngi* and *Pi. longiflocosa* are not.
